# Maternal High-Fat Diet Consumption in Sprague Dawley Rats Compromised the Availability and Altered the Tissue Distribution of Lutein in Neonatal Offspring

**DOI:** 10.3390/metabo13040544

**Published:** 2023-04-11

**Authors:** Yanqi Zhang, Libo Tan

**Affiliations:** Department of Human Nutrition, University of Alabama, Tuscaloosa, AL 35487, USA

**Keywords:** lutein, maternal obesity, neonates, high fat diet, milk

## Abstract

Lutein, the most abundant carotenoid in the infant eye and brain, is critical for their visual and cognitive development. Due to its lipophilic nature, a high adiposity may affect the tissue distribution of lutein. The aim of the study was to determine the impacts of a maternal high-fat diet (HFD) consumption on the status of lutein in the neonatal offspring. Female Sprague Dawley rats (*n* = 6) were fed a normal fat diet (NFD) or a HFD for 8 weeks before mating, and they were switched to an NFD or an HFD containing the same concentration of lutein ester during gestation and lactation. Rat pups (*n* = 7/group/time) were euthanized on postnatal day 2 (P2), P6, P11, and P20 for measuring tissue lutein concentrations. No significant difference in maternal lutein intake was found between the two groups. At both P6 and P11, a significantly lower lutein concentration was noted in the milk samples separated from the stomach of HFD pups than the concentration in the samples from the NFD pups; the HFD group showed a significantly lower lutein concentration in the liver. At P11, the HFD pups exhibited a significantly lower lutein concentration in the eye, brain, and brown adipose tissue accompanied with a significantly higher lutein concentration and mass in the visceral white adipose tissue. The study was the first to provide evidence that maternal HFD consumption resulted in a compromised availability and altered distribution of lutein in the neonatal offspring.

## 1. Introduction

Lutein, a lipophilic phytochemical belonging to the large class of plant pigments known as carotenoids, is a potent scavenger of free radicals due to its extended number of conjugated double bonds [1]. As a powerful antioxidant, lutein plays an important role in the health of infants and preferentially accumulates in the eye and the brain [2,3]. In the eye, lutein is one of the main components of the macular pigment, filtering blue light and protecting the macula from light-initiated oxidative damage [4]. Infants have a significantly higher concentration of lutein in the eye than adults due to the lack of enzymes that convert lutein into other isomers [5]. Meanwhile, emerging evidence demonstrated lutein to be the most predominant carotenoid in the infant brain, accounting for 60% of the total carotenoids there, although only 12% of dietary carotenoids are lutein, indicating a preference for lutein in the infant brain [6]. A variety of evidence showed that the lutein concentration in the brain was positively correlated with concentrations of several neurotransmitters involved in neuron proliferation and maturation, neurite growth, and synaptic formation in human infants [7,8]. Moreover, lutein is the predominant carotenoid in breast milk, emphasizing its importance for infant health and development [9].

Lutein cannot be synthesized in the body and must be obtained from the diet [10]. Therefore, breastfed infants must rely on breast milk to maintain an optimal status of this crucial bio-compound. However, the availability and tissue status of lutein in infants may be compromised by maternal overweightness/obesity, the rate of which in pregnant women in the U.S. has reached 48%. Previous studies showed that maternal overweightness/obesity was associated with a lower concentration of lipophilic nutrients, such as vitamin A, β-carotene, lycopene, and lutein + zeaxanthin, in breast milk [11,12]. Moreover, lutein accumulates in fatty tissues (i.e., the liver and adipose tissue) for long-term storage. It has been hypothesized that the enlarged adipose tissue in obesity may act as a sink to sequester more lipophilic compounds, resulting in lower amounts in circulation and/or in target organs [13]. Such hypotheses were supported for a few lipophilic nutrients in previous studies that reported a significantly lower circulating concentration of vitamin A, vitamin D, or lycopene in adult obese humans and rodents compared with their normal-weight counterparts [14,15,16]. A negative association between body mass index (BMI) and the level of serum α-carotene, β-carotene, β-cryptoxanthin, and lutein/zeaxanthin was observed in young adults [17,18]. Previous epidemiological and observational studies also indicated a negative relationship between the macular pigment optical density and the fat mass in adults [19,20]. Despite these previous findings, no research has addressed this question yet: Does maternal overweightness/obesity and the resultant increased neonatal adiposity affect the availability and status of lutein in the offspring [21,22,23]?

Therefore, the aim of the present study was to determine the impact of maternal high-fat diet consumption in Sprague Dawley rats on the breast milk lutein concentration and the tissue distribution of lutein in the suckling and weanling offspring. It was hypothesized that offspring from mothers consuming a high-fat diet (HFD) would have a compromised lutein status, as exhibited by a significantly lower concentration of lutein in various organs, compared to those from mothers consuming a normal-fat diet (NFD).

## 2. Materials and Methods

### 2.1. Animal Experiment

**Figure 1:**
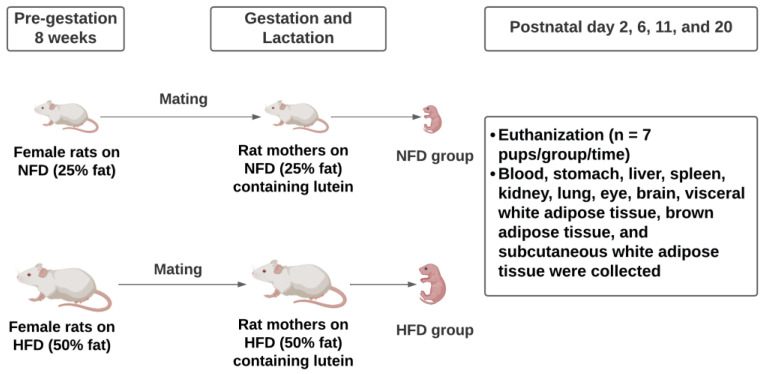
Schematic diagram of study procedures.

The procedure for this experiment was approved by the Institutional Animal Care and Use Committee of the University of Alabama (Protocol number: 20-02-3361). The schematic diagram of the study design is shown in Figure 1. Three-week-old female Sprague Dawley rats (*n* = 6) were purchased from Charles River Laboratories (Wilmington, MA, USA). Rats were housed individually with a 12-h light/dark cycle with free access to food and water. After a 7 day acclimation, rats were randomized to either a purified NFD (25% kcal from fat) or a purified HFD (50% kcal from fat) containing no lutein. Eight weeks later, they were mated with male Sprague Dawley rats of the same age purchased from Charles River.

When male rats were removed from the cages, female rats in the NFD and the HFD group started to consume either an NFD or an HFD, both containing lutein ester at the concentration of 9.8 g/4145 kcal (Table 1). Since rats eat for calories, lutein was formulated into the diets based on total calories. Xangold^®^ lutein ester (30% OLV EU) was a generous gift from BASF (Florham, NJ, USA) and sent to Research Diets, Inc. (New Brunswick, NJ, USA) for diet production. Maternal rats consumed the lutein-containing diets through gestation (~3 weeks) and lactation (3 weeks). Daily food intake and weekly body weight gain of the rat mothers were recorded.

On postnatal day 2 (P2), P6, P11, and P20, rat pups (*n* = 7/group/time) were randomly selected from the litters in each group, weighed, and euthanized with isoflurane. Blood from vena cava, stomach, liver, spleen, kidney, eye, brain, visceral white adipose tissue (VWAT; white adipose tissue surrounding the intra-abdominal organs), interscapular brown adipose tissue (BAT), and subcutaneous white adipose tissue (SWAT) were collected. Blood samples were centrifuged at 1500× *g* for 20 min at 4 °C to obtain serum, and samples were stored at −80 °C; tissue samples were snap-frozen in liquid nitrogen and stored at −80 °C until analysis.

### 2.2. Analysis of Lutein Concentration in Serum, Milk, and Tissues

The concentration of lutein in the serum, milk separated from rat pups’ stomach, and all the other collected tissues was analyzed with ultra-performance liquid chromatography (UPLC) with a photodiode array detector and HSS T3 (1.8 μm, 2.1 mm × 100 mm) column (Acquity UPLC System; Waters, Milford, MA, USA) using an adaption of previously reported methods [24]. For the serum, 0.8 mL of the sample was incubated with 4 mL of 100% ethanol at room temperature for an hour for lipid extraction. For milk samples and tissues, 0.6 g of samples were weighed, homogenized, and incubated with 4 mL of 100% ethanol at room temperature for an hour. An amount of 1.2 mL of 5% (*w*/*v*) potassium hydroxide was added to the sample and incubated in a water bath at 60 °C for 45 min. After cooling down, 4 mL of hexane containing 0.1% butylated hydroxytoluene and 2 mL of deionized H_2_O were added. The upper phase was collected after 15 min of centrifugation and dried under nitrogen. Retinyl acetate (Sigma-Aldrich, St. Louis, MO, USA) was used as the internal standard. The dried sample was rinsed with hexane and reconstituted with 100 µL of acetonitrile: methanol (85:15, *v*/*v*). Possible precipitation was removed by centrifugation. Ten µL of the final sample were injected onto the HSS T3 column for analysis.

### 2.3. Statistical Analysis

Data are reported as mean ± standard deviation (SD). Body weight, tissue weight, and serum and tissue lutein concentration were compared between groups at individual time points using Student’s *t*-test. All data analysis was conducted using GraphPad Prism software (San Diego, CA, USA); *p* < 0.05 was considered statistically significant.

## 3. Results

### 3.1. Dietary Intake, Body Weight, and Adiposity

**Figure 2:**
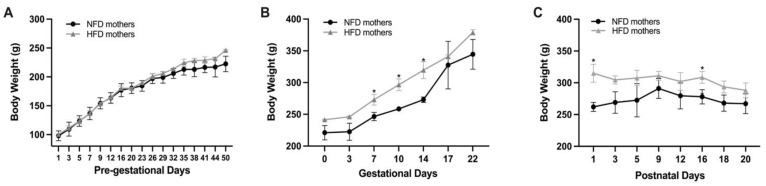
Maternal body weight before gestation (**A**), during gestation (**B**), and during lactation (**C**). Bars show mean ± SD, *n* = 7 per group. Significant difference between groups was indicated by *, *p* < 0.05.

Based on the record of daily food intake during pregnancy and lactation, the daily lutein intake of rat mothers in the groups was not significantly different (Pregnancy: 0.06 ± 0.008 vs. 0.05 ± 0.003 g/d; Lactation: 0.11 ± 0.03 vs. 0.10 ± 0.03 g/d; *p* > 0.05). Rats consuming the HFD started to exhibit a higher body weight than rats consuming the NFD after starting the diet for four weeks (Figure 2A). During gestation and lactation, rat mothers consuming the HFD showed a significantly higher body weight than the NFD mothers on gestational day 7, 10, and 14 and on P1 and P16 (*p* < 0.05; Figure 2B,C).

**Figure 3:**
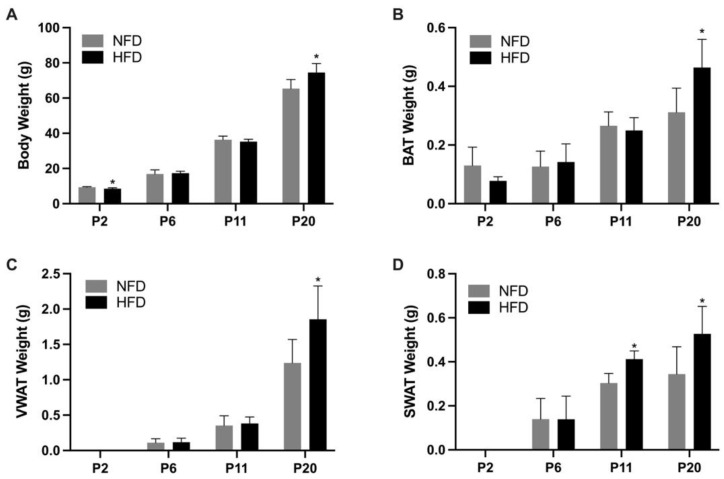
Body weight (**A**), brown adipose tissue weight (**B**), visceral white adipose tissue weight (**C**), and subcutaneous white adipose tissue weight (**D**) of rat pups at postnatal day 2, postnatal day 6, postnatal day 11, and postnatal day 20. Bars show mean ± SD, *n* = 7 per group. Significant difference between groups was indicated by *, *p* < 0.05. At P2, visceral white adipose tissue and subcutaneous white adipose tissue samples were not adequate for analysis, and therefore, data are missing.

The body weight and adiposity of rat pups are shown in Figure 3. At P2, a significantly higher body weight was noted in pups in the NFD group than the HFD group (9.36 ± 0.32 vs. 8.57 ± 0.45 g, *p* < 0.05). The HFD group started to show higher adiposity from P11, as demonstrated by the significantly higher SWAT weight (0.41 ± 0.04 g vs. 0.30 ± 0.04 g, *p* < 0.05). At P20, the HFD group exhibited significantly higher body weight, VWAT weight, BAT weight, and SWAT weight than the NFD group (BW: 74.57 ± 5.06 g vs. 65.33 ± 5.20 g, *p* < 0.05; VWAT weight: 1.85 ± 0.47 g vs. 1.24 ± 0.33 g, *p* < 0.05; BAT weight: 0.46 ± 0.10 vs. 0.31 ± 0.08 g, *p* < 0.05; SWAT weight: 0.53 ± 0.13 vs. 0.34 ± 0.13 g, *p* < 0.05).

### 3.2. Milk and Serum Lutein Concentration in Suckling Rats (P2, P6, and P11)

**Figure 4:**
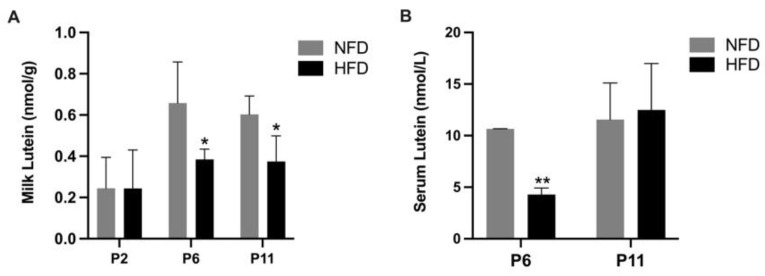
Concentrations of lutein in milk separated from the stomach of neonatal rats (**A**) and in the serum of neonatal rats (**B**). Bars show mean ± SD, *n* = 7 per group. Significant differences between groups were indicated by *, *p* < 0.05 and **, *p* < 0.01. At P2, serum samples were not adequate for analysis, and therefore, data are missing.

The concentration of lutein in milk obtained from the stomach of suckling rat pups and in the serum of those pups are shown in Figure 4. At P6 and P11, the milk lutein concentration was significantly lower in the HFD group than the NFD group (P6: 0.38 ± 0.05 vs. 0.66 ± 0.20 nmol/g, *p* < 0.05; P11: 0.37 ± 0.12 vs. 0.60 ± 0.09 nmol/g, *p* < 0.05). At P6, a significantly lower serum lutein concentration was found in the HFD group compared to the NFD group (P6: 4.30 ± 0.64 vs. 10.66 ± 0.01 nmol/L, *p* < 0.01). At P2, serum samples were not adequate for the analysis.

### 3.3. Tissue Lutein Concentration and Mass in Suckling Rats (P6 and P11)

**Figure 5:**
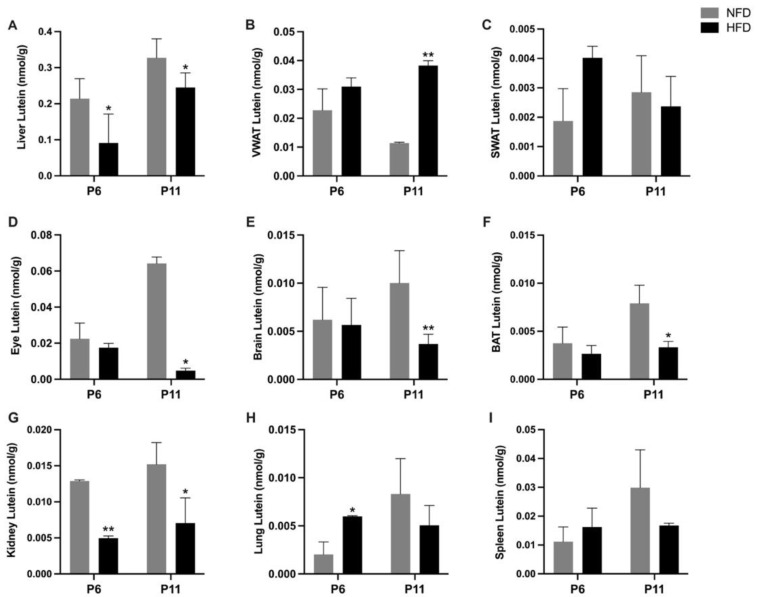
Concentration of lutein in the liver (**A**), visceral white adipose tissue (**B**), subcutaneous white adipose tissue (**C**), eye (**D**), brain (**E**), brown adipose tissue (**F**), kidney (**G**), lung (**H**), and spleen (**I**) of rat pups at postnatal day 6 and postnatal day 11. Bars show mean ± SD, *n* = 7 per group. Significant difference between groups was indicated by *, *p* < 0.05 and **, *p* < 0.01. At P2, tissue samples were not adequate for analysis, and therefore, data are missing.

The lutein concentration in various tissues in suckling rat pups is shown in Figure 5. Most tissue samples collected at P2 were not adequate for the analysis, and therefore, P2 data were not included. At P6 and P11, the HFD group showed a significantly lower lutein concentration in the liver compared to the NFD group (P6: 0.09 ± 0.08 vs. 0.21 ± 0.05 nmol/g, *p* < 0.05; P11: 0.25 ± 0.04 vs. 0.33 ± 0.05 nmol/g, *p* < 0.05). At P6, the HFD group showed a significantly higher lutein concentration in the lung but a significantly lower lutein concentration in the kidney than the NFD group (lung: 0.006 ± 0.00007 nmol/g vs. 0.002 ± 0.001 nmol/g, *p* < 0.05; kidney: 0.005 ± 0.0002 vs. 0.013 ± 0.0003 nmol/g, *p* < 0.05). At P11, the HFD group exhibited a ~3.5 times higher lutein concentration in the VWAT than the NFD group (0.038 ± 0.002 vs. 0.011 ± 0.00004 nmol/g, *p* < 0.01). Notably, the lutein concentration in the eye, brain, BAT, and kidney of rat pups in the HFD group were all significantly lower than those in the NFD group at P11 (eye: 0.005 ± 0.001 vs. 0.064 ± 0.004 nmol/g, *p* < 0.01; brain: 0.004 ± 0.001 vs. 0.01 ± 0.003 nmol/g, *p* < 0.01; BAT: 0.003 ± 0.0006 vs. 0.008 ± 0.002 nmol/g, *p* < 0.05; kidney: 0.007 ± 0.004 vs. 0.02 ± 0.003 nmol/g, *p* < 0.05).

**Figure 6:**
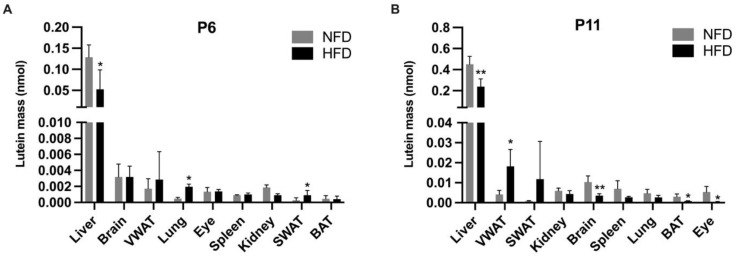
Lutein mass in the tissues of rat pups at postnatal day 6 (**A**) and postnatal day 11 (**B**). Tissues were ranked from the highest to the lowest according to the lutein mass in the high fat diet group. Bars show mean ± SD, *n* = 7 per group. Significant differences between groups were indicated by *, *p* < 0.05 and **, *p* < 0.01. At P2, tissue samples were not adequate for analysis, and therefore, data missing.

The total lutein mass in the tissues was calculated as lutein concentration × tissue weight and is shown in Figure 6. At P6, pups in the HFD group possessed a significantly lower lutein mass in the liver (0.05 ± 0.05 nmol vs. 0.13 ± 0.03 nmol, *p* < 0.05) but a significantly higher lutein mass in the lung (0.002 ± 0.0004 vs. 0.0005 ± 0.0004 nmol, *p* < 0.05) and the SWAT (0.0009 ± 0.0006 vs. 0.0002 ± 0.0003 nmol, *p* < 0.05) compared with the NFD group. At P11, the HFD group exhibited a significantly higher lutein mass in the VWAT (0.02 ± 0.008 vs. 0.004 ± 0.002 nmol, *p* < 0.05) but a significantly lower mass in the liver (0.24 ± 0.07 vs. 0.45 ± 0.08 nmol, *p* < 0.01), brain (0.004 ± 0.001 vs. 0.01 ± 0.003 nmol, *p* < 0.01), BAT (0.001 ± 0.00004 vs. 0.003 ± 0.001 nmol, *p* < 0.05), and eye (0.0005 ± 0.0001 vs. 0.003 ± 0.003 nmol, *p* < 0.05) than the NFD group.

### 3.4. Tissue Lutein Concentration and Mass in Weanling Rats (P20)

**Figure 7:**
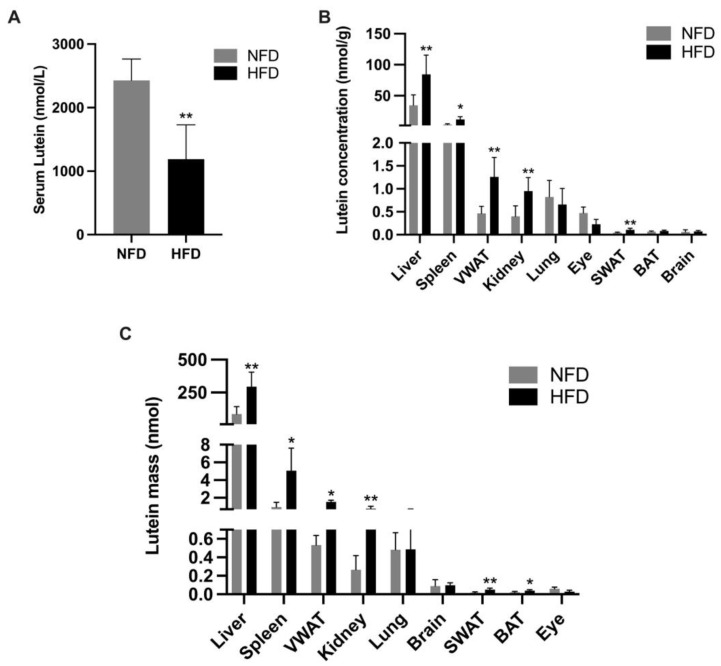
Serum lutein concentration (**A**), tissue lutein concentration (**B**), and tissue lutein mass (**C**) of rat pups at postnatal day 20. Bars show mean ± SD, *n* = 7 per group. Significant differences between groups were indicated by *, *p* < 0.05 and **, *p* < 0.01.

Rat pups started to consume their diet from around P18, which distinguished the pups euthanized at P20 from those euthanized at P2, P6, and P11. It was not feasible to figure out how much of the diet each pup euthanized at P20 consumed since the litter and the mother stayed in the same cage. Therefore, the lutein intake between the two groups cannot be compared, and the P20 data was analyzed separately (Figure 7). At P20, the HFD group exhibited a significantly lower lutein concentration in the serum (Figure 7A; 1189.88 ± 538.15 vs. 2428.64 ± 338.41 nmol/g, *p* < 0.01) but a significantly higher concentration in the liver (84.41 ± 31.15 vs. 34.44 ± 16.82 nmol/g, *p* < 0.01), spleen (11.79 ± 4.49 vs. 3.40 ± 1.42 nmol/g, *p* < 0.05), VWAT (1.27 ± 0.42 vs. 0.46 ± 0.15 nmol/g, *p* < 0.01), kidney (0.95 ± 0.30 vs. 0.40 ± 0.23 nmol/g, *p* < 0.01), and SWAT (0.11 ± 0.03 vs. 0.045 ± 0.01 nmol/g, *p* < 0.01) than the NFD group (Figure 7B). Lutein mass in tissues was shown in Figure 7C. Similarly to the data on lutein concentration, the HFD group showed a significantly higher lutein mass in the liver (294.62 ± 109.97 nmol vs. 86.97 ± 55.58 nmol, *p* < 0.01), spleen (5.07 ± 2.54 vs. 0.94 ± 0.55 nmol, *p* < 0.05), VWAT (1.52 ± 0.20 vs. 0.51 ± 0.10 nmol, *p* < 0.01), kidney (0.78 ± 0.26 vs. 0.27 ± 0.15 nmol, *p* < 0.01), SWAT (0.05 ± 0.016 vs. 0.019 ± 0.008 nmol, *p* < 0.01), and BAT (0.04 ± 0.009 vs. 0.02 ± 0.007 nmol, *p* < 0.05) than the NFD group.

## 4. Discussion

The current study is the first that determined the impact of maternal diet-induced obesity on lutein status in the offspring.

The concentration of lutein in the milk produced by mothers consuming the HFD was found to be ~1.7 times lower than that of the NFD group in spite of the fact that their daily lutein intake was slightly higher. The finding is consistent with previous cross-sectional studies that noted a significantly negative association between maternal BMI and lutein concentration in breast milk [11,12,25]. Lutein is delivered to tissues by lipoproteins upon hydrolysis by lipoprotein lipase (LPL) [26]. On the one hand, it was reported that obese subjects have higher LPL catalytic activity in the adipose tissue, which may lead to a higher lutein uptake by the adipose tissue and, thus, a reduced delivery to the mammary tissue [27,28]. On the other hand, it was hypothesized that obesity-related inflammation suppresses mammary LPL via inflammatory cytokines, such as tumor necrosis factor (TNF)- α, which may contribute to less fatty acid transfer to the milk, as observed in a study by Walker et al. [29]. It is plausible to hypothesize that the mechanism also contributes to the lower transfer of lutein to the milk. In addition, being overweight or obese is associated with elevated levels of oxidative stress. Lutein, as an antioxidant, may be used at a higher extent in selected tissues in overweight and obese mothers, and less is delivered to mammary glands [11,30]. Although more research is required to elucidate the mechanism, current results suggest the potential need for pregnant and lactating mothers who are overweight or obese to increase their lutein intake so that a comparable level of lutein in breast milk can be achieved as in mothers with a normal body weight.

Despite no significant difference in lutein intake, we showed that maternal HFD consumption was associated with compromised lutein status in the suckling rat pups. Specifically, at both P6 and P11, the HFD group showed a significantly lower lutein concentration and mass in the liver, the main storage organ for lutein. At P6, the serum lutein concentration was ~2.5 times lower in the HFD pups than in the NFD pups. At P11, the HFD pups exhibited a significantly lower lutein concentration in the eye, brain, kidney, and BAT, accompanied with a significantly higher lutein concentration and mass in the VWAT. Remarkably, the lutein concentration in the eyes of the HFD pups was ~13 times lower than that in the eyes of the NFD group. Although the lower concentration of lutein in the selected organs in HFD pups may be partly due to the lower concentration of lutein in the milk they received, the accompanying higher lutein concentration in the VWAT proved an altered tissue distribution of lutein. At P11, in NFD pups, the order of lutein concentration in different tissues from the highest to the lowest was liver > eye > spleen > VWAT > kidney > brain > lung > BAT > SWAT while in HFD pups, it was liver > VWAT > spleen > kidney > lung > eye > brain > BAT > SWAT. As for the order of tissue lutein mass, at P11, in NFD pups, it was liver > brain > spleen > kidney > eye > lung > VWAT > BAT > SWAT while in HFD pups, it was liver > VWAT > SWAT > kidney > brain > spleen > lung > BAT > eye. Comparing the two groups, again, it is obvious that maternal HFD consumption is associated with an altered distribution of lutein in the WAT versus in other organs, especially in the eye and the brain.

It is worth noting that not only was the total mass of lutein in the VWAT significantly higher in the HFD pups, which supported the hypothesis that the enlarged adipose tissue may sequester more lipophilic nutrients, but the lutein concentration in the VWAT was also significantly higher [13]. It may be related to the altered expression of xanthophyll metabolizing enzymes and/or transport proteins in the adipose tissue. For example, the expression of CD36, a membrane glycoprotein and a mediator of lutein uptake in adipocytes, was reported to be higher in the adipocytes in HFD-induced obese mice [31,32,33]. Future molecular analysis is needed to determine the expression of other proteins involved in lutein transport and metabolism in neonatal tissues under maternal obesity, such as LDL receptor (LDLR), scavenger receptor type-BI (SR-BI), β-carotene oxygenase 2 (BCO2), and StAR-related lipid transfer domain containing 3 (StARD3) [34].

As mentioned, the data on lutein concentration and mass at P20 (Figure 7) were not included in our main analysis (Figure 4, Figure 5 and Figure 6). According to Figure 7, rat pups in the HFD group had significantly higher lutein concentration and mass in the liver, spleen, VWAT, kidney, and SWAT. It is plausible that HFD pups ate more or had a better absorption of lutein due to the high-fat content in their food. However, it is very interesting to note that even though HFD pups had an increased lutein store in the liver and adipose tissue and a higher concentration in a few other organs, the serum concentration was significantly lower and the concentration in the eye and the brain was not higher than that in the NFD pups. This also implies that a high adiposity might alter the tissue distribution of lutein. Although there were higher lutein stores in the liver and the adipose tissue in HFD pups, excess lutein was not readily mobilized to be present in the circulation or available to the eye and the brain.

The present study possesses several strengths. First, by using the mother–milk–neonate triad model and analyzing all the key neonatal organs, we obtained a comprehensive picture of the influence of maternal HFD consumption on the lutein status in the offspring. We acknowledge that the number of maternal rats was small. Although the sample size was determined by power analysis, future studies should consider a larger size. Maternal measures should also be included to establish the maternal-neonatal correlation in given outcomes. Secondly, the addition of lutein to the maternal diet mimicked the most common approach of lutein consumption in pregnant and lactating women. Since the maternal rats were on a lutein-depleted NFD/HFD for 8 weeks before switching to the lutein-containing NFD/HFD, a high dose of lutein was incorporated in the diets to ensure that adequate lutein would be passed to the rat pups and that lutein in neonatal tissues could be detected. The daily lutein consumption by maternal rats was much lower than the no observed-adverse-effect level (NOAEL) for lutein/zeaxanthin concentrate, which was determined to be 400 mg/kg BW/day in a previous report [35]. The milk lutein concentrations (Figure 4A) were similar to those reported for rhesus macaques or human breastmilk with healthy maternal fruit and vegetable intake, indicating that the neonatal rats did not overconsume lutein [36,37].

It must be pointed out that the tissue metabolism and accumulation of lutein is different in rodents than it is in primates because the major xanthophyll cleavage enzyme BCO2 is inactive in primates [38]. Among mammals, the accumulation of high levels of xanthophyll carotenoids in the retinal and many other extrahepatic tissues is only found in primates [38]. That explains the much lower lutein concentrations in neonatal rat tissues in the current study compared to those reported for breastfed infant rhesus macaques [36]. However, we believe that the observations in the current model are still meaningful. Sprague Dawley rats are among the most used and economical models for diet-induced obesity and were also commonly used for studying the health benefits of lutein [39,40,41]. The findings of this exploratory study warrant further investigation in BCO2 knockout rodent models or in primates.

## 5. Conclusions and Future Directions

To conclude, it was found that breast milk received by neonatal rats in the HFD group contained a lower concentration of lutein compared to the milk from the NFD group. Moreover, maternal HFD consumption was associated with a lower lutein concentration and mass in its target organs in the neonatal offspring. The study adds to the limited evidence that maternal overweightness/obesity is associated with an altered tissue distribution of lipophilic nutrients in the offspring, which compromised the nutrient availability in key organs. It suggests the need for future research to determine if pregnant or lactating women who are overweight/obese should have a higher intake of those nutrients than women with a normal body weight. Furthermore, kinetic studies using isotopically labeled lutein, as well as molecular analysis, are suggested to obtain a comprehensive understanding of how maternal obesity alters lutein dynamics in the offspring at both the whole-body and the cellular level.

## Figures and Tables

**Table 1:** Diet composition of normal- and high-fat purified diets with or without lutein.

	Normal Fat Diet ^a^		Normal Fat Diet + Lutein ^b^		High-Fat Diet ^c^		High-Fat Diet + Lutein ^d^	
	g	kcal	g	kcal	g	kcal	g	kcal
Ingredient								
**Lutein (Xangold, 30%)**	**0**	**0**	**9.8**	**88**	**0**	**0**	**9.8**	**88**
Casein	200	800	200	800	200	800	200	800
L-Cystine	3	12	3	12	3	12	3	12
Corn Starch	353.8	1415	353.8	1415	101.2	405	101.2	405
Maltodextrin 10	125	500	125	500	125	500	125	500
Sucrose	68.8	275	68.8	275	68.8	275	68.8	275
Cellulose, BW200	50	0	50	0	50	0	50	0
Soybean Oil	25	225	25	225	25	225	25	225
Lard	87.7	789	87.7	789	200	1800	200	1800
Mineral Mix S10026	10	0	10	0	10	0	10	0
DiCalcium Phosphate	13	0	13	0	13	0	13	0
Calcium Carbonate	5.5	0	5.5	0	5.5	0	5.5	0
Potassium Citrate, 1 H_2_O	16.5	0	16.5	0	16.5	0	16.5	0
Vitamin Mix V10001	10	40	10	40	10	40	10	40
Choline Bitartrate	2	0	2	0	2	0	2	0
Food color	0.05	0	0.05	0	0.05	0	0.05	0
Total	970.35	4057	980.15	4145	830.05	4057	839.85	4145
	g/kg	Kcal%	g/kg	Kcal%	g/kg	Kcal%	g/kg	Kcal%
Fat	120	25	110	27	270	50	270	51
Carbohydrate	570	30	570	30	370	29	360	29
Protein	210	20	210	20	240	20	240	20
Total	-	100	-	100	-	100	-	100
kcal/g	4.2	-	4.2	-	4.9	-	4.9	-

Formulation details are provided in grams, g, and kilocalories, kcal. ^a^ Research Diets, Inc. (D19120501), 25% fat diet. ^b^ Research Diets, Inc. (D20022104), 25% fat diet with 9.8 g lutein/4145 kcal. ^c^ Research Diets, Inc. (New Brunswick, NJ, USA) (D19120502), 50% fat diet. ^d^ Research Diets, Inc. (New Brunswick, NJ, USA) (D20022105), 50% fat diet with 9.8 g lutein/4145 kcal.

## Data Availability

Due to privacy or ethical restrictions, the data presented in this study are available on request from the corresponding author.

## Abbreviations

brown adipose tissue, BAT; β-carotene oxygenase 2, BCO2; high fat diet, HFD; LDL receptor, LDLR; lipoprotein lipase, LPL; normal fat diet, NFD; retinopathy of prematurity, ROP; scavenger receptor type-BI, SR-BI; StAR related lipid transfer domain containing 3, StARD3; subcutaneous white adipose tissue, SWAT; ultra-performance liquid chromatography system, UPLC; visceral white adipose tissue, VWAT.

## References

[1] Becerra M.O., Contreras L.M., Lo M.H., Díaz J.M., Herrera G.C. (2020). Lutein as a functional food ingredient: Stability and bioavailability. J. Funct. Foods.

[2] Vishwanathan R., Kuchan M.J., Sen S., Johnson E.J. (2014). Lutein and preterm infants with decreased concentrations of brain carotenoids. J. Pediatr. Gastroenterol. Nutr..

[3] Haber N., Heuberger R. (2013). The effect of lutein and zeaxanthin on premature infant eye development. Infant Child Adolesc. Nutr..

[4] Landrum J.T., Bone R.A. (2001). Lutein, zeaxanthin, and the macular pigment. Arch. Biochem. Biophys..

[5] Henriksen B.S., Chan G., Hoffman R.O., Sharifzadeh M., Ermakov I.V., Gellermann W., Bernstein P.S. (2013). Interrelationships between maternal carotenoid status and newborn infant macular pigment optical density and carotenoid status. Investig. Ophthalmol. Vis. Sci..

[6] Jeon S., Neuringer M., Johnson E.E., Kuchan M.J., Pereira S.L., Johnson E.J., Erdman J.W. (2017). Effect of carotenoid supplemented formula on carotenoid bioaccumulation in tissues of infant Rhesus Macaques: A pilot study focused on lutein. Nutrients.

[7] Giampietri M., Lorenzoni F., Moscuzza F., Boldrini A., Ghirri P. (2016). Lutein and neurodevelopment in preterm infants. Front. Neurosci..

[8] Tanprasertsuk J., Li B., Bernstein P.S., Vishwanathan R., Johnson M.A., Poon L., Johnson E.J. (2016). Relationship between concentrations of lutein and StARD3 among pediatric and geriatric human brain tissue. PLoS ONE.

[9] Lipkie T.E., Morrow A.L., Jouni Z.E., McMahon R.J., Ferruzzi M.G. (2015). Longitudinal survey of carotenoids in human milk from urban cohorts in China, Mexico, and the USA. PLoS ONE.

[10] Perrone S., Tei M., Longini M., Buonocore G. (2016). The multiple facets of lutein: A call for further investigation in the perinatal period. Oxidative Med. Cell. Longev..

[11] Zielinska M.A., Hamulka J., Wesolowska A. (2019). Carotenoid content in breastmilk in the 3rd and 6th month of lactation and its associations with maternal dietary intake and anthropometric characteristics. Nutrients.

[12] Xue Y., Campos-Giménez E., Redeuil K.M., Lévèques A., Actis-Goretta L., Vinyes-Pares G., Zhang Y., Wang P., Thakkar S.K. (2017). Concentrations of carotenoids and tocopherols in breast milk from urban Chinese mothers and their associations with maternal characteristics: A cross-sectional study. Nutrients.

[13] Albuquerque M.N., Diniz Ada S., Arruda I.K. (2016). Elevated serum retinol and low beta-carotene but not alpha-tocopherol concentrations are associated with dyslipidemia in Brazilian adolescents. J. Nutr. Sci. Vitaminol..

[14] Carrelli A., Bucovsky M., Horst R., Cremers S., Zhang C., Bessler M., Schrope B., Evanko J., Blanco J., Silverberg S.J. (2017). Vitamin D storage in adipose tissue of obese and normal weight women. J. Bone Miner. Res..

[15] Chai W., Conroy S.M., Maskarinec G., Franke A.A., Pagano I.S., Cooney R.V. (2010). Associations between obesity and serum lipid-soluble micronutrients among premenopausal women. Nutr. Res..

[16] Senkus K.E., Tan L., Crowe-White K.M. (2020). Systemic and adipose tissue redox status in Sprague-Dawley rats fed normal- and high-fat diets supplemented with lycopene. J. Med. Food.

[17] Han G.M., Soliman G.A., Meza J.L., Islam K.M., Watanabe-Galloway S. (2016). The influence of BMI on the association between serum lycopene and the metabolic syndrome. Br. J. Nutr..

[18] Andersen L.F., Jacobs D.R., Gross M.D., Schreiner P.J., Dale Williams O., Lee D.H. (2006). Longitudinal associations between body mass index and serum carotenoids: The CARDIA study. Br. J. Nutr..

[19] Hammond B.R., Ciulla T.A., Snodderly D.M. (2002). Macular pigment density is reduced in obese subjects. Investig. Ophthalmol. Vis. Sci..

[20] Kirby M.L., Beatty S., Stack J., Harrison M., Greene I., McBrinn S., Carroll P., Nolan J.M. (2011). Changes in macular pigment optical density and serum concentrations of lutein and zeaxanthin in response to weight loss. Br. J. Nutr..

[21] Vahratian A. (2009). Prevalence of overweight and obesity among women of childbearing age: Results from the 2002 National Survey of Family Growth. Matern. Child Health J..

[22] Chen C., Xu X., Yan Y. (2018). Estimated global overweight and obesity burden in pregnant women based on panel data model. PLoS ONE.

[23] Gaillard R. (2015). Maternal obesity during pregnancy and cardiovascular development and disease in the offspring. Eur. J. Epidemiol..

[24] Sheshappa M.B., Ranganathan A., Bhatiwada N., Talahalli R.R., Vallikannan B. (2015). Dietary Components Affect the plasma and tissue levels of lutein in aged rats with lutein deficiency—A repeated gavage and dietary study. J. Food Sci..

[25] Yang C., Zhao A., Ren Z., Zhang J., Wang P., Zhang Y. (2022). Vitamin A nutritional status of urban lactating Chinese women and its associated factors. Nutrients.

[26] Parker R.S. (1996). Absorption, metabolism, and transport of carotenoids. FASEB J..

[27] Kern P.A., Ong J.M., Saffari B., Carty J. (1990). The effects of weight loss on the activity and expression of adipose-tissue lipoprotein lipase in very obese humans. N. Engl. J. Med..

[28] Wang H., Eckel R.H. (2009). Lipoprotein lipase: From gene to obesity. Am. J. Physiol. Endocrinol. Metab..

[29] Walker R.E., Harvatine K.J., Ross A.C., Wagner E.A., Riddle S.W., Gernand A.D., Nommsen-Rivers L.A. (2022). Fatty acid transfer from blood to milk is disrupted in mothers with low milk production, obesity, and inflammation. J. Nutr..

[30] Keaney J.F., Larson M.G., Vasan R.S., Wilson P.W., Lipinska I., Corey D., Massaro J.M., Sutherland P., Vita J.A., Benjamin E.J. (2003). Obesity and systemic oxidative stress: Clinical correlates of oxidative stress in the Framingham Study. Arterioscler. Thromb. Vasc. Biol..

[31] Moussa M., Gouranton E., Gleize B., Yazidi C.E., Niot I., Besnard P., Borel P., Landrier J.F. (2011). CD36 is involved in lycopene and lutein uptake by adipocytes and adipose tissue cultures. Mol. Nutr. Food Res..

[32] Shyam R., Vachali P., Gorusupudi A., Nelson K., Bernstein P.S. (2017). All three human scavenger receptor class B proteins can bind and transport all three macular xanthophyll carotenoids. Arch. Biochem. Biophys..

[33] Luo X., Li Y., Yang P., Chen Y., Wei L., Yu T., Xia J., Ruan X.Z., Zhao L., Chen Y. (2020). Obesity induces preadipocyte CD36 expression promoting inflammation via the disruption of lysosomal calcium homeostasis and lysosome function. eBioMed.

[34] Bohn T., Desmarchelier C., Dragsted L.O., Nielsen C.S., Stahl W., Rühl R., Keijer J., Borel P. (2017). Host-related factors explaining interindividual variability of carotenoid bioavailability and tissue concentrations in humans. Mol. Nutr. Food Res..

[35] Ravikrishnan R., Rusia S., Ilamurugan G., Salunkhe U., Deshpande J., Shankaranarayanan J., Shankaranarayana M.L., Soni M.G. (2011). Safety assessment of lutein and zeaxanthin (Lutemax 2020): Subchronic toxicity and mutagenicity studies. Food Chem. Toxicol..

[36] Jeon S., Ranard K.M., Neuringer M., Johnson E.E., Renner L., Kuchan M.J., Pereira S.L., Johnson E.J., Erdman Jr J.W. (2018). Lutein is differentially deposited across brain regions following formula or breast feeding of infant rhesus macaques. J. Nutr..

[37] Xu X., Zhao X., Berde Y., Low Y.L., Kuchan M.J. (2019). Milk and plasma lutein and zeaxanthin concentrations in Chinese breast-feeding mother-infant dyads with healthy maternal fruit and vegetable intake. J. Am. Coll. Nutr..

[38] Li B., Vachali P.P., Gorusupudi A., Shen Z., Sharifzadeh H., Besch B.M., Nelson K., Horvath M.M., Frederick J.M., Baehr W. (2014). Inactivity of human β, β-carotene-9′, 10′-dioxygenase (BCO2) underlies retinal accumulation of the human macular carotenoid pigment. Proc. Natl. Acad. Sci. USA.

[39] Kamil A., Smith D.E., Blumberg J.B., Astete C., Sabliov C., Oliver Chen C.Y. (2016). Bioavailability and biodistribution of nanodelivered lutein. Food Chem..

[40] Kim J.K., Park S.U. (2016). Current results on the potential health benefits of lutein. EXCLI J..

[41] Koushan K., Rusovici R., Li W., Ferguson L.R., Chalam K.V. (2013). The role of lutein in eye-related disease. Nutrients.

